# Training of Youth

**Published:** 1882-10

**Authors:** J. B. Chandler


### TRAINING OF YOUTH.

J. B. CHANDLER.

If a man buys a farm and proposes to stock it, he looks over the place and decides how many horses, cows and oxen it will properly support, and also how many he can attend to, and his judgment is considered good or bad according to the outcome of this decision. Should he make an error, however, it can be easily corrected—the butcher on the one hand, or the market to purchase more on the other.

There are questions of much more importance than this which arise when a man decides to enter into married life, which we fear (from what we see around us of misery and crime in youth) are seldom recognized. Many persons look upon marriage and the gathering around them a large family of children as the entire business of life, and indeed a duty which they owe to Providence—and upon Providence they throw the whole responsibility of the birth, feeding and training of the children. The legal and physical power to get married and bring into the world children is undisputed, even if the parents' position and habits of life are such as promise no training for the offspring beyond that which will lead to the almshouse, or the prison after criminal disturbance of the public peace.

Yet a step further up in the social scale and we find families of children whose parents, having ample means to provide for them, are too indolent or careless to give that early training, that groundwork which must emanate from home, which will insure the safe passage through the vices and temptations which beset youth. Money and influence may cover up for a time the early crimes, but the career of such children too frequently ends in the convict's cell.

How little the fond mother who spoils her children can realize what the uncorrected fault may lead to—the petty theft, the uncontrolled temper, and, worst of all, the deliberate lie. The embryo thief may be manifested in the first; the murderer in the second, and all crimes are concealed in the last. At a tender age children are sent to a public school and brought into direct contact with all classes—those who are utterly devoid of any home-training become the teachers in vice for those whose weak or careless parents have prepared the ground for its reception by lax or spasmodic rule.

Beyond the school comes the almost entire abandonment of the apprentice system, the last chance the boy might have for the correction of his faulty training, and the result is marked in the figures I am about to give, taken from a report of a penal institution of some importance in this country, as being the first to try what is known as the solitary system of imprisonment.

The directors of the Eastern Penitentiary of Pennsylvania annually publish a report of the affairs of the institution for the preceding year, and those reports contain carefully prepared tables from which may be gathered statistics of great value to those who interest themselves in the great problem of human happiness, and what leads to or detracts therefrom.

The tables for 1881 show that the number of convicts sent to that institution during the year, was 433. "Of these 270 were under thirty years of age; Of these 229 were under twenty-seven; Of these again, 181 were under twenty-five, and there were no less than 101 who had not passed their twenty-first year—some of these last being mere boys."

Now let us not forget in considering these figures, that the institution is a penitentiary to which only those convicted of great crimes are sent, not a penal institution for the correction of boys and girls.

From another table we learn the sex and occupation of the criminals. "Of the 433 prisoners received, 423 were males, there being but ten women; and of the males only ninety-eight had any regular occupations; fifty-four had learned trades in a regular way; forty-four had "picked up" trades; and 325 *never learned any trade.*" That is to say, more than three-quarters of the number of convicts to this prison had no trade nor any steady employment for earning a living.

These figures are startling, showing as they do, that nearly *one-quarter* of the convictions of a year to a penitentiary should be of children (to speak legally), boys who have not reached an age to be entirely emancipated from the control of parents or those to whom they should have been apprenticed.

There would seem to be something gradually undermining the whole moral structure of society. Is it beyond our reach? Laws, strenuous ones too, have been passed for the protection of youth from the temptations of the vicious, yet they are comparatively dead letters. Boys are a large element in the patronage of pool shops, rum mills, brothels and other places of vice, and hence, the result is shown in the statistics of the prison, that boys contribute largely towards filling the cells.

Much might be written upon this subject, and possibly with good results in the shape of a plea for the speedy restoration of the old apprentice system, which has become almost obsolete through the action of trade unions. Again, arguments might be brought forward, based upon statistics, as to the peculiar religious training, if any, of a majority of the convicts and its good or bad effects, if either existed.

"As the twig is bent the tree is inclined." Let me then ask the attention of those who take upon themselves the responsibility of being parents, to the weight of that responsibility. Shall their children graduate from their school at home into a college of vice to be followed by handcuffs and a cell? There is something more in the responsibility of a parent than the furnishing of food and clothing.

The Good Book tells us that "the sin of the parent shall be visited upon the children unto the third and fourth generation." It may be just possible that the children may not be the only sufferers for the crimes which the proper care of a parent might have prevented.

